# Ifosfamide-Induced Encephalopathy Successfully Prevented by Methylene Blue: A Pediatric Case Report and Review of the Literature

**DOI:** 10.7759/cureus.40213

**Published:** 2023-06-10

**Authors:** Yu Furui, Kazutoshi Komori, Takashi Kurata, Kazuo Sakashita

**Affiliations:** 1 Department of Hematology and Oncology, Nagano Children's Hospital, Azumino, JPN

**Keywords:** methylene blue treatment, ifosfamide-induced encephalopathy, nicotinamide adenine dinucleotide hydride, thiamine, chloroacetaldehyde, alkylating agent, ewing's sarcoma

## Abstract

Ifosfamide, which is widely used as a chemotherapeutic agent in various kinds of malignancies, sometimes causes neurotoxicity known as ifosfamide-induced encephalopathy (IIE). Herein, we report the case of a three-year-old girl who developed IIE during chemotherapy for Ewing's sarcoma and was treated with methylene blue as a prophylactic agent for IIE, after which she continued with ifosfamide and completed treatment without IIE recurrence. This case suggests that methylene blue may be effective in preventing the recurrence of IIE in pediatric patients. Further studies, including clinical trials, are needed to confirm the efficacy and safety of methylene blue in pediatric patients.

## Introduction

Ifosfamide is an alkylating agent used to treat a wide range of malignancies, including Ewing's sarcoma and various other types of sarcoma and hematologic tumors. Although its most well-known side effects are hemorrhagic cystitis and renal dysfunction, neurotoxicity (known as ifosfamide-induced encephalopathy (IIE)) is also an important side effect. The prevalence of IIE has been reported to be 10%-30% [1-4], and it occurs less frequently in pediatric patients than in adults [5,6]. The most frequently observed neurological symptoms in children with IIE include impaired consciousness, cerebellar ataxia, transient asthenia, urinary incontinence, cranial nerve dysfunction, and seizures [7]. IIE occurs within a week after initiation of ifosfamide, and neurological symptoms are usually transient and resolve within 48-72 hours of onset [8]. However, prolonged neurological symptoms [9] and death [10] have also been reported. The mechanism of action of IIE is thought to be that chloroethylamine, a metabolite of ifosfamide, inhibits the mitochondrial respiratory chain, disrupting the intracellular nicotinamide adenine dinucleotide (NAD)/nicotinamide adenine dinucleotide hydride (NADH) balance, leading to the accumulation of NADH and preventing the dehydrogenation of the neurotoxic chloroacetaldehyde (CAA) [11]. Risk factors for the development of IIE have been reported, including the concomitant use of cisplatin or carboplatin, which leads to renal dysfunction, anti-emetics such as aprepitant, a CYP3A4 inhibitor, opioids, antihistamines, sedatives, and hypoalbuminemia [3,5,12-14].

No treatment is necessary if the patient with IIE recovers spontaneously; however, some cases require anticonvulsants, and there are reports that methylene blue and thiamine are effective as treatment options for the acute phase of the disease [1,15]. Although there is no established strategy for subsequent chemotherapy, including the re-administration of ifosfamide in patients with developing IIE, methylene blue has been reported to be effective in preventing the development of IIE. However, there have been only a few reports of pediatric cases. Herein, we report the case of a three-year-old girl with Ewing's sarcoma who developed IIE during chemotherapy and was successfully prevented from recurrence with methylene blue.

## Case presentation

**Figure 1:**
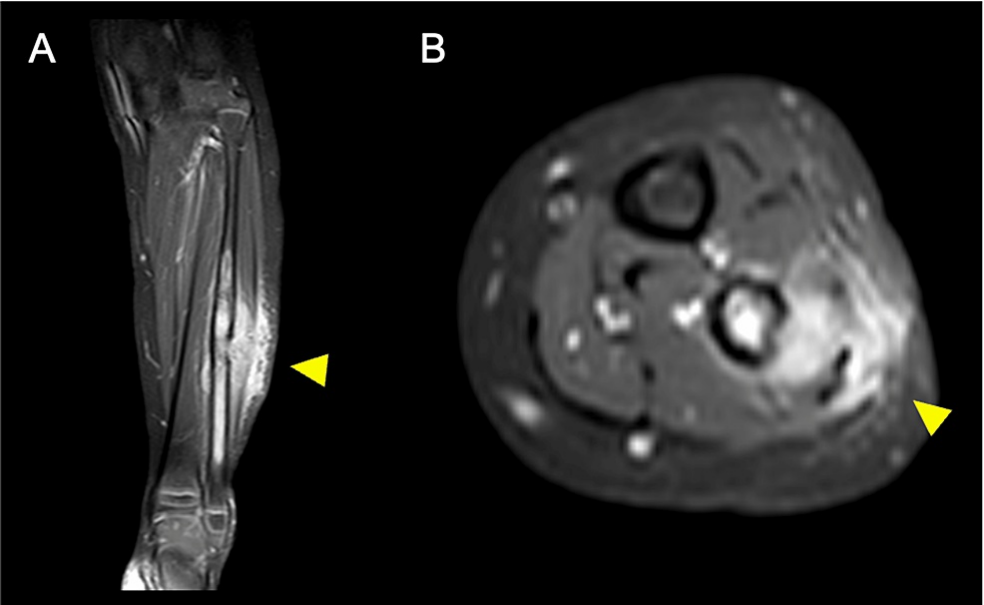
Contrast-enhanced fat-suppressed T1-weighted image of the left lower leg There is a contrast area from the diaphysis of the left fibula to the distal end of the diaphysis. (A) Coronal plane; (B) Axial plane

A three-year-old girl presented to the hospital with swelling and pain in her left lower leg. Imaging studies revealed a suspicious extramedullary bulging neoplastic lesion (Figures 1A, 1B); thus, a tissue biopsy was performed.

**Figure 2:**
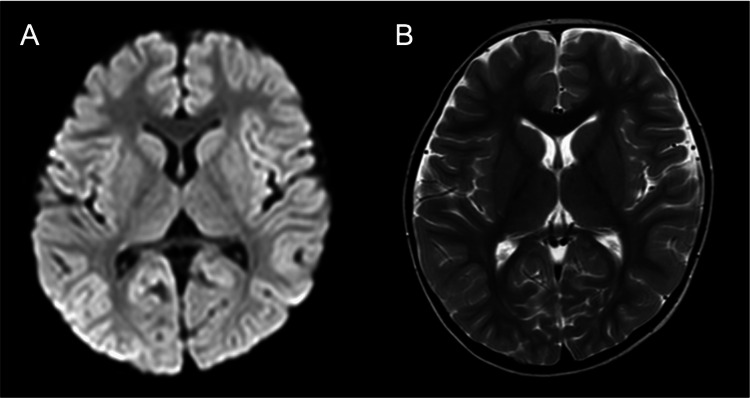
Head MRI There were no abnormal findings on the head MRI. (A) Diffusion-weighted image; (B) T2-weighted image

The patient was referred to our hospital with a diagnosis of Ewing's sarcoma. No distant metastasis was found, and the patient was started on multi-agent chemotherapy comprising vincristine, doxorubicin, cyclophosphamide, ifosfamide, and etoposide (VDC-IE). We administered 1.8 g/m2 of ifosfamide over one hour for five days and 2-mercaptoethanesulfonate (Mesna) to prevent hemorrhagic cystitis. Chemotherapy resulted in tumor shrinkage, and after six cycles of VDC-IE, a left peroneal extensive resection was performed. Proton beam therapy (50.4 Gy/28 Fr) was administered as adjuvant postoperative irradiation with chemotherapy. During the fifth cycle of IE, immediately after the end of day one of ifosfamide administration, the patient presented with impaired consciousness, bilateral eye deviation, and limb weakness, which was determined to be a non-convulsive seizure. The neurological symptoms improved quickly after the administration of midazolam as an anticonvulsant. Blood tests, head MRI (Figures 2A, 2B), electroencephalography (EEG), and lumbar puncture were performed to investigate the cause.

There were no abnormal findings on the head MRI, electroencephalogram (EEG), or cerebrospinal fluid (CSF). After ruling out hypoglycemia, electrolyte abnormalities, meningitis, posterior reversible encephalopathy syndrome (PRES), and other conditions through various tests, the clinical course led to a diagnosis of ifosfamide encephalopathy (IIE). Neurotoxicity was evaluated as grade four per the National Cancer Institute's Common Toxicity Criteria (NCI-CTC) classification [16]. Chemotherapy was temporarily interrupted; however, since the resumption of treatment was desirable for the radical cure of the primary disease, we decided to continue treatment with methylene blue to prevent IIE based on previous reports [17-19]. Since methylene blue is used off-label in Japan, it was used with the consent of the parents after Ethics Committee approval was obtained. Based on the Euro Ewing 2012 protocol [20], methylene blue was administered at a dose of 1 mg/kg/dose 24 hours before ifosfamide administration and every six hours during ifosfamide therapy. The formulation was dissolved in 5% dextrose and administered intravenously over 10 minutes. The ifosfamide administration time was extended from one hour to three hours. Considering that fosaprepitant affects ifosfamide metabolites through its CYP3A4 inhibitory effect, the drug was not used during subsequent IE therapy. Prophylaxis was continued during the ifosfamide treatment period for three cycles of IE therapy until the end of treatment, and the entire course of treatment was completed as planned with no neurological symptoms suggestive of IIE relapse. One year and four months after the end of treatment, the patient remains in remission.

## Discussion

We report a pediatric patient who developed IIE and was treated with a regimen including ifosfamide with methylene blue as prophylaxis for the development of IIE. Methylene blue is known to cause serious side effects such as shock, anaphylaxis, hemolytic anemia, and renal failure. It can also cause common side effects such as hyperhidrosis, skin discoloration, taste abnormalities, nausea, dizziness, and extremity pain. However, in this case, the only side effect seen was transient urine discoloration. This case suggests that methylene blue might be effective and safe for preventing IIE in pediatric patients.

There are two possible approaches to the subsequent treatment of patients who develop IIE. One is to switch therapy to an alternate alkylating agent such as cyclophosphamide [5]. However, it is unclear whether the change from ifosfamide to cyclophosphamide guarantees therapeutic efficacy [21]. Another is the prevention of the development of IIE by methylene blue, as it suppresses mitochondrial respiratory chain inhibition by chloroethylamine, oxidizes NADH, and reduces the formation of CAA by inhibiting the activity of monoamine oxidase. This mechanism may have led to therapeutic and prophylactic effects on the development of IIE [11]. Furthermore, its efficacy in preventing IIE in adults has been reported [1,22]. Pelgrims et al. [1] reported that, among twelve patients with IIE, three received prophylactic administration of methylene blue. Of these, one patient had no recurrence and two had a recurrence, but the grade of IIE was reduced from the initial onset. Similarly, Turner et al. [22] reported that two of four patients with IIE received methylene blue prophylaxis, and one patient had no recurrence while the other had a recurrence with a reduced grade of neurologic toxicity. These findings suggest that methylene blue prophylaxis may not only prevent IIE recurrence but also reduce the severity of neurologic toxicity when recurrence occurs. However, there are only a few case reports on its use as a prophylactic agent for pediatric-onset IIE [17-19]. Table 1 shows the pediatric cases who received methylene blue for prophylactic treatment.

**Table 1:** Summary of cases using methylene blue for prevention of IIE in pediatric patients IFO: ifosfamide; IIE: ifosfamide-induced encephalopathy; NCI-CTC: National Cancer Institute's Common Toxicity Criteria; N/A: data not available

Author	Tumor diagnosis	Age	Sex	IFO dose (g/m^2^/day)	IIE grade (NCI-CTC)	Treatment for IIE	Prophylaxis dose of methyleneblue	Adverse event	Outcome
Dufour et al., 2006 [17]	N/A	N/A	N/A	N/A	N/A	N/A	N/A	N/A	No recurrence
	N/A	N/A	N/A	N/A	N/A	N/A	N/A	N/A	No recurrence
Ames et al., 2010 [19]	Osteosarcoma	16	Male	2.8	4	Observation	50 mg/dose q8h	N/A	No recurrence
	Osteosarcoma	7	Female	2.8	4	Methylene blue	2.4 mg/kg/dose q4h	N/A	Recurrence
Sarbay et al., 2021 [18]	Osteosarcoma	15	Female	3	4	Anticonvulsant	50 mg/dose q6h	N/A	No recurrence
Current case	Ewing's sarcoma	3	Female	1.8	4	Anticonvulsant	1.0 mg/kg/dose q6h	No	No recurrence

Five of the six patients did not experience an IIE recurrence. These cases suggest that methylene blue may be safe and effective for preventing IIE, even in pediatric cases. However, the administration time and dosage varied, and these are considered issues for the future.

There are some caveats to this case report. First, the efficacy of methylene blue as a prophylactic agent against IIE cannot be determined based on a single case. The duration of ifosfamide administration was extended from one hour to three hours, and fosaprepitant was discontinued, making it unclear whether methylene blue alone prevented IIE. Second, while no serious adverse reactions have been reported in pediatric patients treated with methylene blue, and none were observed in this case, the number of reported cases is small, and further studies are needed to fully evaluate its safety in this population. Third, in Japan, methylene blue is currently indicated only for toxic methemoglobinemia and is not approved for the treatment or prevention of IIE. Therefore, additional studies, including clinical trials, are required to establish the efficacy and safety of methylene blue as a prophylactic agent against IIE and expand its indication.

## Conclusions

In conclusion, methylene blue could be useful in preventing IIE recurrence in children, and the appropriate use of methylene blue may allow patients to complete chemotherapy after recovering from IIE. Further intervention studies are needed to determine the best method of administering methylene blue.
